# Diagnostic validity of methods for assessment of swallowing sounds: a systematic review^☆^

**DOI:** 10.1016/j.bjorl.2017.12.008

**Published:** 2018-02-03

**Authors:** Karinna Veríssimo Meira Taveira, Rosane Sampaio Santos, Bianca Lopes Cavalcante de Leão, José Stechman Neto, Leandro Pernambuco, Letícia Korb da Silva, Graziela De Luca Canto, André Luís Porporatti

**Affiliations:** a Universidade Federal do Rio Grande do Norte (UFRN), Departamento de Morfologia, Centro de Biociências, Natal, RN, Brazil; b Universidade Tuiuti do Paraná (UTP), Programa de Pós-graduação em Distúrbios da Comunicação, Curitiba, PR, Brazil; c Universidade Tuiuti do Paraná (UTP), Departamento de Odontologia, Curitiba, PR, Brazil; d Universidade Federal da Paraíba (UFPB), Departamento de Fonoaudiologia, João Pessoa, PB, Brazil; e Instituto de Educação Luterana de Santa Catarina, Departamento de Fonoaudiologia, Joinville, SC, Brazil; f Universidade Federal de Santa Catarina (UFSC), Departamento de Odontologia, Brazilian Centre for Evidence-based Research, Florianópolis, SC, Brazil; g University of Alberta, Faculty of Medicine and Dentistry, School of Dentistry, Alberta, Canada

**Keywords:** Deglutition, Deglutition disorders, Diagnosis, Review, Deglutição, Distúrbios de deglutição, Diagnóstico, Revisão

## Abstract

**Introduction:**

Oropharyngeal dysphagia is a highly prevalent comorbidity in neurological patients and presents a serious health threat, which may lead to outcomes of aspiration pneumonia, ranging from hospitalization to death. This assessment proposes a non-invasive, acoustic-based method to differentiate between individuals with and without signals of penetration and aspiration.

**Objective:**

This systematic review evaluated the diagnostic validity of different methods for assessment of swallowing sounds, when compared to videofluroscopy swallowing study to detect oropharyngeal dysphagia.

**Methods:**

Articles in which the primary objective was to evaluate the accuracy of swallowing sounds were searched in five electronic databases with no language or time limitations. Accuracy measurements described in the studies were transformed to construct receiver operating characteristic curves and forest plots with the aid of Review Manager v. 5.2 (The Nordic Cochrane Centre, Copenhagen, Denmark). The methodology of the selected studies was evaluated using the Quality Assessment Tool for Diagnostic Accuracy Studies-2.

**Results:**

The final electronic search revealed 554 records, however only 3 studies met the inclusion criteria. The accuracy values (area under the curve) were 0.94 for microphone, 0.80 for doppler, and 0.60 for stethoscope.

**Conclusion:**

Based on limited evidence and low methodological quality because few studies were included, with a small sample size, from all index testes found for this systematic review, doppler showed excellent diagnostic accuracy for the discrimination of swallowing sounds, whereas microphone-reported good accuracy discrimination of swallowing sounds of dysphagic patients and stethoscope showed best screening test.

## Resumo

**Introdução:**

A disfagia orofaríngea é uma comorbidade altamente prevalente em pacientes neurológicos e representa uma séria ameaça à saúde, pode levar a desfechos como pneumonia por aspiração, hospitalização e até morte. A avaliação propõe um método não invasivo, acústico, para diferenciar entre indivíduos com e sem sinais de penetração e aspiração.

**Objetivo:**

Esta revisão sistemática analisou a validade diagnóstica de diferentes métodos para avaliação dos sons de deglutição, quando comparados com a videofluoroscopia da deglutição para detectar disfagia orofaríngea.

**Método:**

Artigos nos quais o objetivo principal era avaliar a acurácia dos sons de deglutição foram pesquisados em cinco bancos de dados eletrônicos sem limitações de idioma ou tempo de publicação. As medidas de acurácia descritas nos estudos foram transformadas para construir curvas ROC (*Receptor Operating Characteristic*) e gráfico em floresta (*forest plot*) com o auxílio do software *Review Manager v. 5.2* (The Nordic Cochrane Centre, Copenhagen, Dinamarca). A metodologia dos estudos selecionados foi avaliada com a ferramenta Avaliação da Qualidade de Estudos de Acurácia de Testes Diagnósticos-2.

**Resultados:**

A busca eletrônica final resultou na identificação de 554 artigos; no entanto, apenas três estudos preencheram os critérios de inclusão. Os valores de acurácia (área abaixo da curva) foram 0,94 para microfone, 0,80 para doppler e 0,60 para estetoscópio.

**Conclusão:**

Baseado nas evidências limitadas e da baixa qualidade metodológica, pois foram poucos os estudos incluídos, e com pequeno tamanho amostral, de todos os testes diagnósticos (*index testes*) encontrados para essa revisão sistemática o doppler mostrou excelente acurácia diagnóstica na discriminação dos sons de deglutição, o microfone demonstrou uma boa acurácia na discriminação dos sons de pacientes disfágicos e o estetoscópio revelou o melhor teste de triagem.

## Introduction

Swallowing is characterized by an intricate neuromuscular mechanism that requires a sequence of biomechanical activities, resulting in the passage of liquids and solids from mouth to stomach, avoiding the airway.^1^, ^2^ Dysphagia may bring serious and potentially fatal health consequences, which negatively impact the well-being, safety, quality of life, and safety of patients.^3^, ^4^ Aspiration is one of the most serious manifestations of oropharyngeal dysphagia, and may be the cause of undernourishment, chest infection, prolonged hospital stay and, lastly, mortality.^5^ Prevalence measurements for dysphagia diverge, depending upon the etiology and patient's age, but estimates as high as 38% for lifetime prevalence have been reported in those over 65-years-old.^6^

To avoid unfavorable health results, detecting dysphagia early is crucial as well as to initiate an early referral for diagnosis and treatment to minimize health threats. The test named Videofluroscopic Swallowing Study (VFSS), which consists of asking a patient to swallow different foods and liquids that contain a radiopaque contrast agent while observed by a trained professional is often considered the standard reference to determine of dysphagia exists.^7^, ^8^, ^9^, ^10^, ^11^ For this test, kinematic X-ray data for physiological swallow impairment and subsequent misdirection of swallowed material^12^, ^13^ are observed by a trained examiner. However, frequent VFSS test repetitions are not recommended due to high radiation exposures.^14^

There is a noninvasive method that has been proposed by acoustic means for swallowing analysis. Microphones and/or accelerometers are used to record breath and swallowing sounds, which are examined using digital signal processing methods. Swallowing sounds have been widely associated with pharyngeal reverberations arising from opening and closing of valves (oropharyngeal, laryngeal and esophageal valves), action of numerous pumps (pharyngeal, esophageal, and respiratory pumps) and vibrations of the vocal tract.^15^

Literature on swallowing sounds to supplement the clinical evaluation of dysphagia has shown promising results.^16^, ^17^ There are no studies correlating the diagnostic accuracy as a method for the detection of swallowing sounds. Based on the above, the aim of this systematic review was to answer the focused question: “What is the diagnostic validity of different methods for assessment of swallowing sounds, when compared to VFSS, for detecting oropharyngeal dysphagia?”

## Methods

### Protocol and registration

PRISMA statement^18^ was used to guide the execution of this systematic review; and the protocol was registered on International Prospective Register of Systematic Reviews (PROSPERO) database (Registration n° CRD42016052771).

### Eligibility criteria

We have included diagnostic validity studies, which used different methods for assessment of swallowing sounds compared to the reference standard: videofluoroscopy (VFSS). Different methods for assessment of swallowing sounds could include ultrasound, acoustic analysis, cervical auscultation, swallowing accelerometers signals, and the Doppler effect. Previous studies from all languages and with no restrictions regarding age, sex and time of publication were included.

### Exclusion criteria

Articles were excluded from review based on the following criteria: (1) Studies in animals; (2) Studies that did not perform ultrasound, acoustic analysis, cervical auscultation, swallowing accelerometers signals or Doppler effect; (3) Studies that did not compare methods of diagnosis of swallowing for both control and dysphagic group with the VFSS reference standard; (4) Studies that did not present validity measurements (sensitivity and specificity) or did not present data enough to calculate them; (5) Reviews, letters, conference, abstract, personal opinions.

### Information sources

A computerized literature search was conducted in five main databases, such as Cochrane, Latin American and Caribbean Health Sciences (LILACS), PubMed (including Medline), Scopus, Web of Science; and three grey literature databases (Google Scholar, OpenGrey, and ProQuest Dissertation and Thesis). More information on the search strategies is provided in Appendix 1. Furthermore, the reference lists of the selected articles were inspected for additional literature. Relevant papers on this topic were also requested from experts in the field. The references were managed and the duplicates hits were removed with the aid of EndNote Basic X7^®^ Software (Thompson Reuters, New York, NY, USA). We conducted all searches on October 8th, 2016. An updated search with the same word combinations for each database above mentioned was performed on January 25th, 2017.

### Study selection

Two independent reviewers (K.V.M.T. and R.S.S.) made the first preselecting cut by screening all articles on title and abstract. Studies which did not appear to meet the eligibility criteria were excluded. Next, they independently screened full texts of this initial set of articles. Any disagreements were resolved through discussion or referral to a third author (B.L.C.L.).

### Data collection process

Data extraction was performed by one author (K.V.M.T.) and checked by a second (R.S.S.). Disagreements were resolved through discussion. A third author (B.L.L.C.L.) became involved, when needed, to make a final decision.

### Data items

The data collected consisted of study authors, year of publication, country, design, mean age and range, sample size, number of patients, number of observations, index test, reference test, description, outcomes, and conclusions. Efforts were made to contact the authors to recover any unpublished data, if the required data were not complete.

### Risk of bias in individual studies

The included studies were assessed for methodological quality using the Quality Assessment Tool for Diagnostic Accuracy Studies (QUADAS-2).^19^ The following four methodological domains were measured for each trial: patient selection, index test, reference standard, flow of patients through the study, and timing of the tests.

Two independent reviewers (K.V.M.T. and R.S.S.) used its critical appraisal criteria to analyze all included articles, scoring each criterion with ‘yes’, ‘no’, or ‘unclear’. Disagreements by discussion with a third author (B.L.C.L.) were made when necessary. Figures of the risk of bias assessment for all included studies were generated with Review Manager 5.3 (RevMan 5.3, The Nordic Cochrane Centre, Copenhagen, Denmark).

### Summary measures

Sensitivity and specificity of the diagnostic tests were the main outcomes evaluated. Positive Predictive Value (PPV), Negative Predictive Value (NPV), Positive Likelihood Ratio (LR+), Negative Likelihood Ratio (LR−), Diagnostic Odds Ratio (DOR), and Youden's index were secondary outcomes. The cutoff values used to interpret these data are presented in Appendix 2.

### Synthesis of results

Cochrane Collaboration guidelines^20^ was used to combine individual results by means of a systematic review, with Restricted Maximum-Likelihood (REML) estimation and the DerSimonian pooled method. All statistical analysis was crude, without adjustment for potential confounders. Some of the required data were not specified in the articles, so we calculated them. Review Manager 5.3 (RevMan 5.3, The Nordic Cochrane Centre, Copenhagen, Denmark) was used to draw Receiver Operating Characteristic (ROC) curves, graphs, and forest plots. Heterogeneity within studies was evaluated either by considering clinical, methodological, and statistical characteristics or by using inconsistency Indexes (*I*^2^), whereas a value greater than 50% was considered an indicator of substantial heterogeneity between studies, and a random effect applied. The significance level was set at 5%.^21^

### Risk of bias across studies

Clinical, methodological, and statistical heterogeneity were explored among studies.

## Results

### Study selection

**Figure 1:**
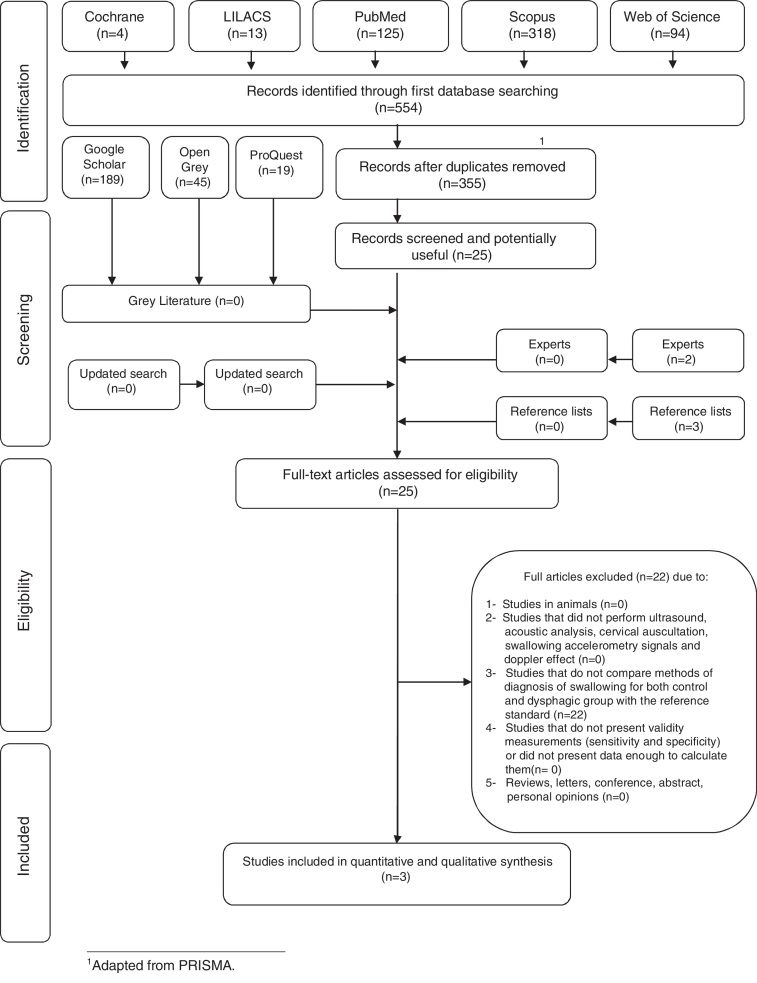
Flow diagram of literature search and selection criteria.^1^

Systematic searches yielded 554 results, as shown in the PRISMA (Fig. 1). After removing the duplicates, a comprehensive evaluation of the 355 abstracts was performed and 330 articles were excluded, resulting in 25 articles for full-text reading. Grey literature search identified 253 studies, where none of the studies were selected. Also, after hand-search of the reference lists and articles provided by experts, no additional studies were included.

Therefore, 25 articles were retrieved for full-text reading. Twenty-two of them were excluded (Appendix 3). Finally, three studies remained and were included in the qualitative synthesis.

### Study characteristic

The three included studies were published in 2004, 2013, and 2015. They were conducted in Brazil,^22^ Japan^23^ and United Kingdom.^24^ The sample size ranged from 10 to 30 healthy patients and 14 to 70 dysphagic patients. The index tests used were microphone^23^ stethoscope with a microphone inserted into tubing at the bifurcation^24^ and sonar Doppler.^22^

The consistencies and viscosities of the material used to execute the reference test also varied. Abdulmassih et al.^22^ used three consistencies: liquid, 70 mL water and 30 mL of 100% barium sulfate; pudding, 70 mL of water, 30 mL of barium sulfate; solids, club social biscuits soaked in barium during the reference test. Jayatilake et al.^23^ used water swallow test to group healthy and 3 mL water mixed 25% barium group dysphagic during the reference test and Leslie et al.^24^ used two consistencies, 3 boluses each of 5, 20 mL thin barium and 5 mL yogurt during the reference test. The liquid bolus volumes in the reference test varied from 3^24^ to 70 mL.^22^ The size of the solid boluses was expressed in club social biscuits soaked in barium. Characteristics of included studies are described in Table 1.

**Table 1:** Summary of descriptive characteristics and outcomes of interest of the included studies (*n* = 3).

Author, year, country	Mean age range (years)	Sample size n° of patients	Sample size n° of observations	Index test	Reference test	Description	Outcome	Conclusion
Abdulmassih et al., 2013, Brazil^18^	46.4 (28–62) healthy	30 healthy	30 healthy	Doppler	VFSS Swallow material: liquid, 70 mL water and 30 mL of 100% barium sulfate; pudding, 70 mL of water, 30 mL of barium sulfate; solids, club social biscuits soaked in barium	Acoustic analysis of swallow	The prevalence in the dynamic evaluation of swallowing VFSS was by changes in the oral phase of swallowing. The analysis of variance of the averages found in each variable – frequency, intensity and duration of swallowing – shows there was a significant correlation when compared to the healthy individual curve.	In patients with SCA, the mean initial frequency, initial intensity, and final intensity were higher and the time and peak frequency were lower, demonstrating a pattern of cricopharyngeal opening very close to that found in normal populations.
	44.9 (28–62) dysphagic	30 dysphagic	30 dysphagic					

Jayatilake et al., 2015, Japan^19^	(22–39) healthy	15 healthy	8 healthy	Microphone	VFSS Swallow material: group healthy, water swallow test; group dysphagic, 3 mL water mixed 25% barium	Real-time swallowing sound-processing algorithm for the automatic screening, quantitative evaluation, and the visualization of swallowing ability	71 dry swallows the automatic swallow recognition algorithm achieved sensitivity 93.9% healthy subjects; algorithm automatically detected all or some of the swallowing events of all the 31 subjects dysphagic, and the overall detection accuracy for the 92 swallowing episodes was 79.3%	Swallowscope can analyze swallowing sounds in realtime and generate quantitative results: the number of swallows and the swallowing duration, which can assist bedside screening, and share them through a cloud-based system. We achieved very good performances in terms of both the positive predictive value and sensitivity.
	68.8 dysphagic	70 dysphagic	31 dysphagic					

Leslie et al., 2004, United Kingdom^20^	72 (24–78) healthy	10 healthy	10 healthy	Stethoscope	VFSS Swallow material: 3 boluses each of 5, 20 mL thin barium and 5 mL yogurt	Acoustic analysis of swallow	Comparison with radiological defined aspiration/penetration yielded 66% specificity, 62% sensitivity, and majority consensus gave 90% specificity, 80% sensitivity for detecting normality of a swallow, when consensus is reached among the raters.	Improving the poor raters would improve the overall accuracy of this technique in predicting abnormality in swallowing. The group consensus correctly identified 17 of the 20 clips so we may speculate that the swallow sound contains audible cues that should in principle permit reliable classification.
	78 (65–90) dysphagic	14 dysphagic	10 dysphagic					

VFSS, Videofluoroscopic Swallowing Study; SCA, Spinocerebellar Ataxia; RSST, Repetitive Saliva Swallowing Test.

### Risk of bias within studies

**Figure 2:**
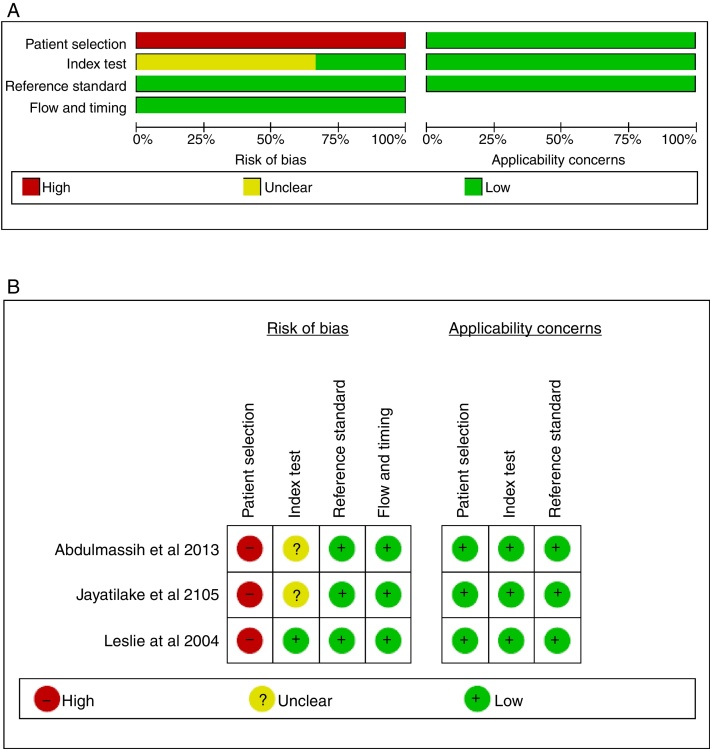
Results from QUADAS-2 study quality assessment (A, risk of bias graph; B, risk of bias summary).

Although no studies fulfilled all criteria of risk of bias, the studies methods were very homogeneous and all possessed low risk of bias for applicability concerns (Appendix 4). For every study, item one of domain one that discuss risk of bias of patient selection was scored as high risk of bias, because each study recruited an experimental sample, without randomization of the enrolled patients. Item one of domain “index test” was scored “unclear” for two studies, because of results of screening or the interpretation of the test. The items reference test, flow and timing for the three included studies were scored “low”. Fig. 2 summarizes QUADAS-2 assessments.

### Results of individual studies

Abdulmassih et al.^22^ evaluated acoustic analysis of swallow on 30 healthy patients and 30 dysphagic patients using a sonar Doppler compared to the VFSS. The analysis of variance of the averages found in each variable – frequency, intensity and duration of swallowing – shows there was a significant correlation when compared to the healthy individual curve.

Jayatilake et al.^23^ evaluated real-time swallowing sound on 8 healthy subjects and 31 dysphagic patients using a microphone compared to the VFSS. 71 dry swallows the automatic swallow recognition algorithm achieved sensitivity 93.9%; algorithm automatically detected all or some of the swallowing events of all the 31 subjects dysphagic, and the overall detection accuracy for the 92 swallowing episodes was 79.3%.

Leslie et al.^24^ evaluated acoustic analysis of swallow on 10 healthy subjects and 10 dysphagic patients using a microphone compared to the VFSS. When the assessors were asked whether the swallow was normal or abnormal, the sensitivity and specificity were low (sensitivity 62%, specificity 66%). When consensus was reached among the raters, the majority consensus gave 90% specificity, 80% sensitivity for detecting swallow normality.

### Synthesis of results

All three articles^22^, ^23^, ^24^ contained enough data to be included in our systematic review. A diagnostic test validity table was constructed using the data extracted from each study (Table 2). In this table, all prevalence and accuracy measurements (sensitivity, specificity, PPV, NPV, LR+, LR−, DOR, and Youden's index) are presented. The total sample size for this systematic review was 117 subjects, 48 healthy subjects and 69 dysphagic patients.

**Table 2:** Diagnostic test validity data (*n* = 3).

Group	Author, year	Dysphasic sample size	Control sample size	Prevalence (%)	Sensitivity (%)	Specificity (%)	PPV	NPV	LR+	LR−	DOR	Youden's index
Doppler/VFSS	Abdulmassih et al., 2013^18^	24	30	50.0^a^	80.0^a^	100^a^	1.00^a^	0.83^a^	∞^a^	0.20^a^	∞^a^	0.80^a^
Microphone/VFSS	Jayatilake et al., 2015^19^	31	8	79.4	93.9	29.1^a^	0.83	0.55^a^	1.32^a^	0.20^a^	0.20^a^	0.23^a^
Stethoscope/VFSS	Leslie et al., 2004^20^	14	10	58.3^a^	62.0	66.0	0.71^a^	0.55^a^	1.82^a^	0.57^a^	3.16^a^	0.28^a^

VFSS, Videofluoroscopic Swallowing Study; PPV, Positive Predictive Value; NPV, Negative Predictive Value; LR+, Positive Likelihood Ratio; LR−, Negative Likehood Ratio; ∞, infinite.

a Data calculated by the authors from information available in the article.

**Figure 3:**
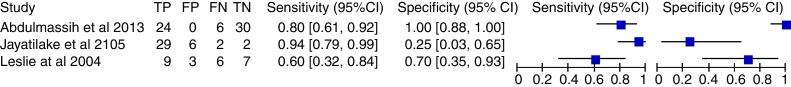
Coupled forest plot of the sensitivity and specificity in videofluroscopic swallowing studies compared and swallow sounds (*n* = 3).

Sensitivity and specificity for different selected studies varied substantially. The diagnostic accuracy (sensitivity, specificity, and 95% Confidence Interval) of each study included in this systematic review is shown in Fig. 3. Sensitivity and specificity for microphone was 94% and 25% (95% CI 0.79–0.99) respectively,^23^ sensitivity and specificity for Doppler was 80% and 100% (95% CI 0.61–0.92) respectively^22^ and sensitivity and specificity for stethoscope was 62% and 66% (95% CI 0.32–0.84) respectively.^24^

The orders of the best diagnostic tests for dysphagic patients were microphone, Doppler and stethoscope. The orders of the best diagnostic tests for healthy patients were Doppler, stethoscope and microphone.

### Additional analysis

**Figure 4:**
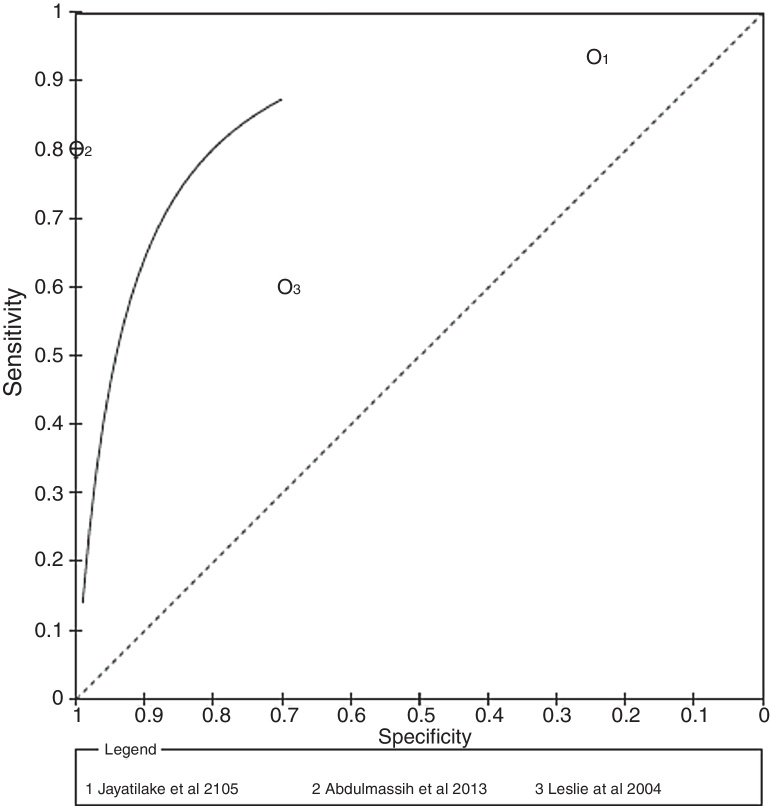
ROC curves of the sensitivity and specificity in videofluroscopic swallowing studies compared and swallow sounds.

We chose to showcase the systematic review results in ROC curves (Fig. 4). Because of differences in the assessment of swallowing sounds methods, no cutoff point measures were justified and thus no threshold effect was possible; therefore, a symmetric curve was applied.

Regarding PPV values, the highest PPV values reported for microphone and Doppler^22^, ^23^ showing that these techniques were able to discriminate swallowing sounds without lesion data 100% of the time. Doppler also reported to have the highest NPV, distinguishing control patients from those with acoustical analysis the swallowing 100% of the time.^22^

Regarding LR values, 3 studies showed LR+ greater than 1.00 for swallowing sounds with stethoscope, microphone and Doppler,^22^, ^23^, ^24^ which means that all methods captured argue for dysphagia.^25^ The highest LR+ value was reported for Doppler (LR+ = ∞)^22^ followed by stethoscope (LR+ = 1.85)^24^ and microphone (LR+ = 1.32)^23^ LR− values closer to 0 were reported for Doppler and microphone,^22^, ^23^ which means a low probability of disease when it is absent in the examination.^25^

Finally, Doppler and stethoscope reported the highest DOR,^22^, ^24^ indicating better discriminatory test performance.^26^ The Doppler reported good Youden's Index (0.80).^22^

### Risk of bias across studies

The main methodological limitations across studies were related to poor reporting for Quadas-2 item “risk of bias of patient selection” scored with high risk of bias. Additionally, a poor agreement across the index test's observers was related at two studies, or it was unclear, resulting in a risk of bias of index test.

## Discussion

This systematic review investigated different methods for assessment of swallowing sounds comparing VFSS among patients with oropharyngeal dysphagia. While several non-instrumented screening procedures have been adopted in medical centers worldwide, efforts to develop improved dysphagia screening methods with both high sensitivity and specificity are currently in development. In this systematic review the presented Doppler has good sensitivity and specificity to capture swallowing sounds and can be used as a method of diagnosis of dysphagic patients and healthy subjects, being a inexpensive and non-invasive method in relation to the reference standard VFSS. The presented microphone has high sensitivity and low specificity to capture swallowing sounds and can be used as a method of diagnosis of dysphagic patients, while the presented stethoscope has low sensitivity and low specificity to capture swallowing sounds and can be used as a method of screening of dysphagic patients.

VFSS is a radiologic procedure, whereby subjects ingest small amounts of barium-coated boluses while X-rays penetrate the subject and the resultant images are video-recorded. The VFSS test allows immediate visual inspection; however, it is time-consuming, non-portable and results in some radiation exposure.^27^ Due to radiation exposure, the VFSS procedure is limited in duration and cannot be frequently repeated.^28^ Thus, new techniques need to be developed to help assess the performance of the swallowing mechanism.

Some reproducible characteristic sound patterns have been reported to be heard during auscultation of swallows with a stethoscope,^29^ microphone^30^, ^31^ or accelerometer^30^, ^32^, ^33^ and Doppler.^22^, ^34^

We found only 3 eligible studies with data concerning Doppler, microphone and stethoscope.^22^, ^23^, ^24^ No data were found for accelerometry. The sensitivity and specificity index values of tests varied among the included studies. The differences in these scores probably reflect the method of sounds of swallowing that are captured, placed on the neck to detect cervical sounds generated during the swallow and breath sounds pre- and post-swallow. Microphones and/or accelerometers are used to record breath and swallowing sounds, which are analyzed then using digital signal processing techniques. The research on using swallowing sounds to supplement the clinical evaluation of dysphagia has shown promising results.^16^

The PPV and NPV values confirmed the ROC curve results (Fig. 2). The higher PPV related to Doppler showed a lower risk of false positive results. In addition, a high NPV noticed in Doppler evaluations indicates that there is also a lower risk of under-diagnosis. It is important to emphasize that the prevalence of a disease can affect PPV and NPV values. When prevalence is high, true-positive results are more likely to be found in the population instead of false-positives, increasing the PPV and decreasing the NPV, respectively.^35^ Similarly, the DOR values of index tests reported indicate that Doppler resulted in better discriminatory test performance^22^ and satisfied the criteria required for an excellent diagnostic test. Finally, LR+ and LR− values expressed better diagnostic accuracy for Doppler.^17^ The Doppler reported good Youden's Index (0.80).^22^

To the best of the authors’ knowledge, this is the first systematic review to validate sensitivity and specificity of sounds of swallowing. These values, added to PPV, NPV, LR+, LR−, ROC curve, and Youden's index analyses, were used for diagnostic accuracy.

In this study, the best diagnostic accuracy results were reported when using Doppler for captured the swallowing sound and can be used as a method of evaluation of dysphagic patients and healthy subjects, being a cheap and non-invasive method in relation to the reference standard VFSS.

Some methodological limitations of this review should be considered. First, different methods of catching swallowing sounds were used. Furthermore, 22 studies had to be excluded due to the lack of compared methods of diagnosis of swallowing for both control and dysphagic group with the reference test may be due to exposure to radiation to healthy patients. Finally, regarding the risk of bias from the included studies, no information about blinding was reported by most of the studies. Also, the preponderance of studies failed to report if the standard reference results were interpreted without knowledge of the results of the index test.

Studies that did not compare an index test with the reference test were not included, because only an acceptable reference test can prove the clinical relevance and reduce the risks of both false positive as well as the false-negative findings. Studies lacking comparisons of methods of diagnosis of swallowing for both control and dysphagic group with the reference test were also excluded.

## Conclusion

Based on limited evidence and low methodological quality because few studies were included, with a small sample size, from all index testes found for this systematic review, the Doppler showed excellent diagnostic accuracy on the discrimination of swallowing sounds, whereas the microphone reported good sensitivity for discrimination of swallowing sounds of dysphagic patients and the stethoscope showed best screening test on the discrimination of swallowing sounds. Further studies with different methods for evaluation of swallowing sounds and with more representative samples are fully encouraged. Additional studies on this topic with a paired control group are also recommended.

## Conflicts of interest

The authors declare no conflicts of interest.

## Figures and Tables

**Table:** 

Database	Search (October 8th 2016; updated on January 25th, 2017)
Cochrane	“deglutition” OR “deglutitions” OR “swallowing” OR “swallowings” OR “swallows” OR “swallow” in Title, Abstract, Keywords and videofluoroscopy OR “Videofluoroscopy Swallowing Study” OR VFSS OR “videofluoroscopy study” OR “swallowing videofluoroscopy” OR videofluoroscopic OR “videofluoroscopic swallowing” OR “videofluoroscopic swallowing study” OR fluoroscopy OR fluoroscopies in Title, Abstract, Keywords and ultrasonography OR ultrasound OR ultrasonics OR “duplex doppler ultrasonography” OR “doppler duplex ultrasonography” OR “acoustic analysis” OR “acoustical analysis” OR auscultation OR “cervical auscultation” OR accelerometry OR “swallowing accelerometry signals” OR “swallowing sounds” OR “swallow sounds” OR “signal processing” OR acoustics OR acoustic OR “doppler effect” OR “doppler shift” OR “sonar doppler” OR microphone in Title, Abstract, Keywords in Trials’
LILACS	(tw:(“deglutition” OR “deglutitions” OR “swallowing” OR “swallowings” OR “swallows” OR “swallow”)) AND (tw:(videofluoroscopy OR “videofluoroscopy swallowing study” OR vfss OR “videofluoroscopy study” OR “swallowing videofluoroscopy” OR videofluoroscopic OR “videofluoroscopic swallowing” OR “videofluoroscopic swallowing study” OR fluoroscopy OR fluoroscopies)) AND (tw:(ultrasonography OR ultrasound OR ultrasonics OR “duplex doppler ultrasonography” OR “doppler duplex ultrasonography” OR “acoustic analysis” OR “acoustical analysis” OR auscultation OR “cervical auscultation” OR accelerometry OR “swallowing accelerometry signals” OR “swallowing sounds” OR “swallow sounds” OR “signal processing” OR acoustics OR acoustic OR “doppler effect” OR “doppler shift” OR “sonar doppler” OR microphone))
PubMed	(“deglutition”[MeSH Terms] OR “deglutition”[All Fields] OR “deglutitions”[All Fields] OR “swallowing”[All Fields] OR “swallowings”[All Fields] OR “swallows”[MeSH Terms] OR “swallows”[All Fields] OR “swallow”[All Fields]) AND (videofluoroscopy[All Fields] OR “videofluoroscopy swallowing study”[All Fields] OR VFSS OR “videofluoroscopy study”[All Fields] OR “swallowing videofluoroscopy”[All Fields] OR videofluoroscopic[All Fields] OR “videofluoroscopic swallowing”[All Fields] OR “videofluoroscopic swallowing study”[All Fields] OR fluoroscopy[MeSH Terms] OR fluoroscopy[All Fields] OR fluoroscopies) AND (ultrasonography[Subheading] OR ultrasonography[All Fields] OR ultrasound[All Fields] OR ultrasonography[MeSH Terms] OR ultrasound[All Fields] OR ultrasonics[MeSH Terms] OR ultrasonics[Text Word] OR ultrasonics[All Fields] OR “duplex doppler ultrasonography”[All Fields] OR “doppler duplex ultrasonography”[All Fields] OR “acoustic analysis”[All Fields] OR “acoustical analysis”[All Fields] OR auscultation[MeSH Terms] OR auscultation[All Fields] OR “cervical auscultation”[All Fields] OR accelerometry[MeSH Terms] OR accelerometry[All Fields] OR “swallowing accelerometry signals”[All Fields] OR “swallowing sounds”[All Fields] OR “swallow sounds”[All Fields] OR “signal processing”[All Fields] OR acoustics[MeSH Terms] OR acoustics[All Fields] OR acoustic[All Fields] OR “doppler effect”[MeSH Terms] OR doppler effect[Text Word] OR “doppler effect”[All Fields] OR “doppler shift” OR “shift, doppler” OR “effect, doppler” OR “sonar doppler”[All Fields] OR microphone[All Fields])
Scopus	(TITLE-ABS-KEY(“deglutition” OR “deglutitions” OR “swallowing” OR “swallowings” OR “swallows” OR “swallow”) AND TITLE-ABS-KEY(videofluoroscopy OR “Videofluoroscopy Swallowing Study” OR VFSS OR “videofluoroscopy study” OR “swallowing videofluoroscopy” OR videofluoroscopic OR “videofluoroscopic swallowing” OR “videofluoroscopic swallowing study” OR fluoroscopy OR fluoroscopies) AND TITLE-ABS-KEY(ultrasonography OR ultrasound OR ultrasonics OR “duplex doppler ultrasonography” OR “doppler duplex ultrasonography” OR “acoustic analysis” OR “acoustical analysis” OR auscultation OR “cervical auscultation” OR accelerometry OR “swallowing accelerometry signals” OR “swallowing sounds” OR “swallow sounds” OR “signal processing” OR acoustics OR acoustic OR “doppler effect” OR “doppler shift” OR “sonar doppler” OR microphone))
Web of Science	Tópico:(“deglutition” OR “deglutitions” OR “swallowing” OR “swallowings” OR “swallows” OR “swallow”) ANDTópico: (videofluoroscopy OR “Videofluoroscopy Swallowing Study” OR VFSS OR “videofluoroscopy study” OR “swallowing videofluoroscopy” OR videofluoroscopic OR “videofluoroscopic swallowing” OR “videofluoroscopic swallowing study” OR fluoroscopy OR fluoroscopies)AND Tópico: (ultrasonography OR ultrasound OR ultrasonics OR “duplex doppler ultrasonography” OR “doppler duplex ultrasonography” OR “acoustic analysis” OR “acoustical analysis” OR auscultation OR “cervical auscultation” OR accelerometry OR “swallowing accelerometry signals” OR “swallowing sounds” OR “swallow sounds” OR “signal processing” OR acoustics OR acoustic OR “doppler effect” OR “doppler shift” OR “sonar doppler” OR microphone))
Google Scholar	“deglutition OR swallowing:videofluoroscopy”
Open Grey	Swallowing OR deglutition AND videofluoroscopy AND “acoustic analysis”
ProQuest	Swallowing OR deglutition AND videofluoroscopy AND “acoustic analysis”

**Table:** 

Test indicators	Data analysis	References
DOR	The value of a DOR ranges from 0 to infinity, with higher values indicating better discriminatory test performance. A value of 1 means that a test does not discriminate between patients with the disorder and those without it. Values lower than 1 point to improper test interpretation (more negative tests among the diseased).	Glas et al.^37^
LRs	>1 – diagnostic of interest	McGee^25^
	0 and 1 – against the diagnosis of interest	
	0 – less likely the disease	
	=1 – lack diagnostic valeu	
Sensitivity	80% excellent, 70–80% good, 60–69% fair, <60% poor	No consensus in this regard exists in the literature.
Specificity	90% excellent, 80–90% good, 70–79% fair, <70% poor	No consensus in this regard exists in the literature.
Youden's Index	Youden's Index values close to 1 indicate high accuracy; a value of zero is equivalent to uninformed guessing and indicates that a test has no diagnostic value.	Deeks et al.^38^

**Table:** 

Author, year	Reason for exclusion
Dudik et al., 2016^39^	3
Dudik et al., 2015^40^	3
Dudik et al., 2016^41^	3
Frakking et al., 2016^42^	3
Frakking et al., 2016^43^	3
Golabbakhsh et al., 2014^44^	3
Lee et al., 2006^45^	3
Mérey et al., 2012^46^	3
Morinière et al., 2011^47^	3
Movahedi et al., 2016^48^	3
Nikjoo et al., 2011^32^	3
Reddy et al., 2000^49^	3
Sejdic et al., 2014^50^	3
Seidic et al., 2013^51^	3
Selley et al., 1994^52^	3
Spadotto et al., 2009^53^	3
Spadotto et al., 2008^54^	3
Steele et al., 2013^55^	3
Stroud et al., 2002^56^	3
Tanaka et al., 2012^57^	3
Zoratto et al., 2010^58^	3
Lazareck et al., 2004^59^	3

Exclusion criteria: (1) studies in animals; (2) studies that did not performed ultrasound, acoustic analysis, cervical auscultation, swallowing accelerometry signals and doppler effect; (3) studies that do not compare methods of diagnosis of swallowing for both control and dysphagic group with the reference standard (videofluoroscopy); (4) studies that do not present validity measurements (sensitivity and specificity) or did not present data enough to calculate them; (5) reviews, letters, conference, abstract, personal opinions.

**Table:** 

	Item	Abdulmassih et al., 2013^18^	Jayatilake et al., 2015^19^	Leslie et al., 2004^20^
Domain 1: Patient selection	Was a consecutive or random sample of patients enrolled?	N	N	N
	Was a case-control design avoided?	N	N	N
	Did the study avoid inappropriate exclusions?	Y	Y	Y
	**Could the selection of patients have introduced bias?**	H	H	H
	**Concerns regarding applicability:** Are there concern that the included patients and settings do not match the review question?	L	L	L

Domain 2: Index test	Were the index test results interpreted without knowledge of the results of the reference standard?	U	U	U
	If a threshold was used, was it prespecified?	Y	Y	Y
	**Could the conduct or interpretation of the index test have introduced bias?**	U	U	L
	**Concerns regarding applicability:** Are there concerns that the index test, its conduct, or its interpretation differ from the review question?	L	L	L

Domain 3: Reference standard	Is the reference standard likely to correctly classify the target condition?	Y	Y	Y
	Were the reference standard results interpreted without knowledge of the results of the index test?	U	U	U
	**Could the reference standard, its conduct, or its interpretation have introduced bias?**	L	L	L
	**Concerns regarding applicability:** Are there concerns that the target condition as defined by the reference standard does not match the question?	L	L	L

Domain 4: Flow and timing	Was there an appropriate interval between index test(s) and reference standard?	Y	Y	Y
	Did all patients receive the same reference standard?	Y	Y	Y
	Were all patients included in the analysis?	Y	N	N
	**Could the patient flow have introduced bias?**	L	L	L

Y, yes; N, no; U, unclear; Risk: L, low; H, high; U, unclear.

## References

[1] Logemann J.A. (1984). Evaluation and treatment of swallowing disorders. Nat Student Speech Lang Hear Assoc J.

[2] Miller A.J. (2008). The neurobiology of swallowing and dysphagia. Dev Disabil Res Rev.

[3] Sura L., Madhavan A., Carnaby G., Crary M.A. (2012). Dysphagia in the elderly: management and nutritional considerations. Clin Interv Aging.

[4] Chen P.H., Golub J.S., Hapner E.R., Johns M.M. (2009). Prevalence of perceived dysphagia and quality-of-life impairment in a geriatric population. Dysphagia.

[5] Smithard D.G., O’Neill P.A., Parks C., Morris J. (1996). Complications and outcome after acute stroke. Does dysphagia matter?. Stroke.

[6] Roy N., Stemple J., Merrill R.M., Thomas L. (2007). Dysphagia in the elderly: preliminary evidence of prevalence, risk factors, and socioemotional effects. Ann Otol Rhinol Laryngol.

[7] Martin-Harris B., Brodsky M.B., Michel Y., Castell D.O., Schleicher M., Sandidge J. (2008). MBS measurement tool for swallow impairment – MBSImp: establishing a standard. Dysphagia.

[8] Logemann J.A. (1998).

[9] Jung S.H., Lee L.K., Hong J.B., Han T.R. (2005). Validation of clinical dysphagia scale: based on videofluoroscopic swallowing study. J Korean Acad Rehabil Med.

[10] McCullough G.H.W.R., Rosenbek J.C., Mills R.H., Webb W.G., Ross K.B. (2001). Inter- and intrajudge reliability for videofluoroscopic swallowing evaluation measures. Dysphagia.

[11] Scott A.P.A., Bench J. (1998). A study of interrater reliability when using videofluoroscopy as an assessment of swallowing. Dysphagia.

[12] Ian J.C., Petter J.K. (1999). American gastroenterological association technical reviewon management of oropharyngeal dysphagia. Gastroenterology.

[13] Rugiu M. (2007). Role of videofluoroscopy in evaluation of neurologic dysphagia. Acta Otorhinolaryngol Ital.

[14] Bours G.J., Speyer R., Lemmens J., Limburg M., de Wit R. (2009). Bedside screening tests vs. videofluoroscopy or fibreoptic endoscopic evaluation of swallowing to detect dysphagia in patients with neurological disorders: systematic review. J Adv Nurs.

[15] Cichero J.A., Murdoch B.E. (1998). The physiologic cause of swallowing sounds: answers from heart sounds and vocal tract acoustics. Dysphagia.

[16] Pehlivan M., Yuceyar N., Ertekin C., Celebi G., Ertaş M., Kalayci T. (1996). An electronic device measuring the frequency of spontaneous swallowing: digital phagometer. Dysphagia.

[17] Lazareck L.J., Moussavi Z.M.K. (2004). Classification of normal and dysphagic swallows by acoustical means. IEEE Trans Biomed Eng.

[18] David M.A.L., Alessandro L., Jennifer T., Douglas G.A., The PRISMA Group (2009). Preferred reporting items for systematic reviews and meta-analyses: the PRISMA statement. PLoS Med.

[19] Whiting P.F., Rutjes A.W., Westwood M.E., Mallett S., Deeks J.J., Reitsma J.B. (2011). QUADAS-2: a revised tool for the quality assessment of diagnostic accuracy studies. Ann Intern Med.

[20] Macaskill P.G.C., Deeks J., Harbord R., Takwoingi Y., Collaboration C. (2010). Cochrane handbook for systematic reviews of diagnostic test accuracy version 1.0.

[21] Deeks J, Gatsonis C. The Cochrane Collaboration. Cochrane handbook for systematic reviews of diagnostic test accuracy version 1.0. Available from: http://srdta.cochrane.org/.

[22] Abdulmassih E.M., Teive H.A., Santos R.S. (2013). The evaluation of swallowing in patients with spinocerebellar ataxia and oropharyngeal dysphagia: a comparison study of videofluoroscopic and sonar Doppler. Int Arch Otorhinolaryngol.

[23] Jayatilake D., Ueno T., Teramoto Y., Nakai K., Hidaka K., Ayuzawa S. (2015). Smartphone-based real-time assessment of swallowing ability from the swallowing sound. IEEE J Transl Eng Health Med.

[24] Leslie P., Drinnan M.J., Finn P., Ford G.A., Wilson J.A. (2004). Reliability and validity of cervical auscultation: a controlled comparison using videofluoroscopy. Dysphagia.

[25] McGee S. (2002). Simplifying likelihood ratios. J Gen Intern Med.

[26] Glas A.S.L.J., Prins M.H., Bonsel G.J., Bossuyt P.M. (2003). The diagnostic odds ratio: a single indicator of test performance. J Clin Epidemiol.

[27] Palmer J.B., Kuhlemeier K.V., Tippett D.C., Lynch C. (1993). A protocol for the videofluorographic swallowing study. Dysphagia.

[28] Ramsey D.J.C., Smithard D.G., Kalra L. (2003). Early assessments of dysphagia and aspiration risk in acute stroke patients. Stroke.

[29] Dempsey J.E., Vice F.L., Bosma J.F. (1990). Combination of cervical auscultation and videoradiography in evaluation of oral and pharyngeal dysphagia. Abstr Symp Dysphagia.

[30] Cichero J.A., Murdoch B.E. (2002). Detection of swallowing sounds: methodology revisited. Dysphagia.

[31] Sarraf S.S., Buchel C., Daun R., Lenton L., Moussavi Z. (2012). Detection of swallows with silent aspiration using swallowing and breath sound analysis. Med Biol Eng Comput.

[32] Nikjoo M.S., Steele C.M., Sejdic E., Chau T. (2011). Automatic discrimination between safe and unsafe swallowing using a reputation-based classifier. Biomed Eng Online.

[33] Dudik J.M., Kurosu A., Coyle J.L., Sejdić E. (2015). A comparative analysis of DBSCAN, K-means, and quadratic variation algorithms for automatic identification of swallows from swallowing accelerometry signals. Comput Biol Med.

[34] Lagos H.N., Santos R.S., Abdulmassih E.M., Gallinea L.F., Langone M. (2013). Characterization of swallowing sounds with the use of sonar Doppler in full-term and preterm newborns. Int Arch Otorhinolaryngol.

[35] Last J.M. (1988).

[36] De Luca Canto G., Pachêco-Pereira C., Aydinoz S., Major P.W., Flores-Mir C., Gozal D. (2015). Diagnostic capability of biological markers in assessment of obstructive sleep apnea: a systematic review and meta-analysis. J Clin Sleep Med.

[37] Glas A.S., Lijmer J.G., Prins M.H., Bonsel G.J., Bossuyt P.M. (2003). The diagnostic odds ratio: a single indicator of test performance. J Clin Epidemiol.

[38] Deeks J.J., Bossuyt P., Gatsonis C. (2010). Cochrane handbook for systematic reviews of diagnostic test accuracy version 1.0. The Cochrane Collaboration.

[39] Dudik J.M., Coyle J.L., Ei-Jaroudi A., Sun M.G., Sejdic E. (2016). A matched dual-tree wavelet denoising for tri-axial swallowing vibrations. Biomed Signal Process Control.

[40] Dudik J.M., Kurosu A., Coyle J.L., Sejdic E. (2015). A comparative analysis of DBSCAN, K-means, and quadratic variation algorithms for automatic identification of swallows from swallowing accelerometry signals. Comput Biol Med.

[41] Dudik J.M., Kurosu A., Coyle J.L., Sejdic E. (2016). A statistical analysis of cervical auscultation signals from adults with unsafe airway protection. J Neuroeng Rehabil.

[42] Frakking T., Chang A., O’Grady K., David M., Weir K. (2016). Aspirating and nonaspirating swallow sounds in children: a pilot study. Ann Otol Rhinol Laryngol.

[43] Frakking T.T., Chang A.B., O’Grady K.F., David M., Weir K.A. (2016). Reliability for detecting oropharyngeal aspiration in children using cervical auscultation. Int J Speech Lang Pathol.

[44] Golabbakhsh M., Rajaei A., Derakhshan M., Sadri S., Taheri M., Adibi P. (2014). Automated acoustic analysis in detection of spontaneous swallows in Parkinson's disease. Dysphagia.

[45] Lee J., Blain S., Casas M., Kenny D., Berall G., Chau T. (2006). A radial basis classifier for the automatic detection of aspiration in children with dysphagia. J Neuroeng Rehabil.

[46] Merey C., Kushki A., Sejdic E., Berall G., Chau T. (2012). Quantitative classification of pediatric swallowing through accelerometry. J Neuroeng Rehabil.

[47] Moriniere S., Boiron M., Brunereau L., Beutter P., Patat F. (2011). Pharyngeal swallowing sound profile assessed after partial and total laryngectomy. Dysphagia.

[48] Movahedi F., Kurosu A., Coyle J.L., Perera S., Sejdic E. (2017). Anatomical directional dissimilarities in tri-axial swallowing accelerometry signals. IEEE Trans Neural Syst Rehabil Eng.

[49] Reddy N.P., Katakam A., Gupta V., Unnikrishnan R., Narayanan J., Canilang E.P. (2000). Measurements of acceleration during videofluorographic evaluation of dysphagic patients. Med Eng Phys.

[50] Sejdic E., Dudik J.M., Kurosu A., Jestrovic I., Coyle J.L. (2014). Understanding differences between healthy swallows and penetration-aspiration swallows via compressive sensing of tri-axial swallowing accelerometry signals. Proc SPIE Int Soc Opt Eng.

[51] Sejdic E., Steele C.M., Chau T. (2013). Classification of penetration–aspiration versus healthy swallows using dual-axis swallowing accelerometry signals in dysphagic subjects. IEEE Trans Biomed Eng.

[52] Selley W.G., Ellis R.E., Flack F.C., Bayliss C.R., Chir B., Pearce V.R. (1994). The synchronization of respiration and swallow sounds with videofluoroscopy during swallowing. Dysphagia.

[53] Spadotto A.A., Gatto A.R., Guido R.C., Montagnoli A.N., Cola P.C., Pereira J.C. (2009). Classification of normal swallowing and oropharyngeal dysphagia using wavelet. Appl Math Comput.

[54] Spadotto A.A., Papa J.P., Gatto A.R., Cola P.C., Pereira J.C., Guido R.C. (2008). Denoising swallowing sound to improve the evaluator's qualitative analysis. Comput Electr Eng.

[55] Steele C.M., Sejdić E., Chau T. (2013). Noninvasive detection of thin-liquid aspiration using dual-axis swallowing accelerometry. Dysphagia.

[56] Stroud A.E., Lawrie B.W., Wiles C.M. (2002). Inter- and intra-rater reliability of cervical auscultation to detect aspiration in patients with dysphagia. Clin Rehabil.

[57] Tanaka N., Nohara K., Okuno K., Kotani Y., Okazaki H., Matsumura M. (2012). Development of a swallowing frequency meter using a laryngeal microphone. J Oral Rehabil.

[58] Zoratto D.C.B., Chau T., Steele C.M. (2010). Hyolaryngeal excursion as the physiological source of swallowing accelerometry signals. Physiol Meas.

[59] Lazareck L., Moussavi Z. (2004). Classification of normal and dysphagic swallows by acoustical means. IEEE Trans Biomed Eng.

